# Impact of Intracellular Concentrations on Metabolic Drug-Drug Interaction Studies

**DOI:** 10.1208/s12248-019-0344-8

**Published:** 2019-06-18

**Authors:** Andrea Treyer, Mohammed Ullah, Neil Parrott, Birgit Molitor, Stephen Fowler, Per Artursson

**Affiliations:** 1 Department of Pharmacy, Uppsala University, Box 580, SE-751 23 Uppsala, Sweden; 2 Roche Pharmaceutical Research and Early Development, Roche Innovation Center Basel, Basel, Switzerland; 3 Science for Life Laboratory Drug Discovery and Development platform (SciLifelab DDD-P), Uppsala, Sweden; 4 Uppsala University Drug Optimization and Pharmaceutical Profiling Platform (UDOPP), Uppsala University, Uppsala, Sweden

**Keywords:** drug-drug interaction, intracellular bioavailability, physiologically based pharmacokinetic modeling, scaling factor, unbound drug concentrations

## Abstract

Accurate prediction of drug-drug interactions (DDI) is a challenging task in drug discovery and development. It requires determination of enzyme inhibition *in vitro* which is highly system-dependent for many compounds. The aim of this study was to investigate whether the determination of intracellular unbound concentrations in primary human hepatocytes can be used to bridge discrepancies between results obtained using human liver microsomes and hepatocytes. Specifically, we investigated if Kp_uu_ could reconcile differences in CYP enzyme inhibition values (K_i_ or IC_50_). Firstly, our methodology for determination of Kp_uu_ was optimized for human hepatocytes, using four well-studied reference compounds. Secondly, the methodology was applied to a series of structurally related CYP2C9 inhibitors from a Roche discovery project. Lastly, the Kp_uu_ values of three commonly used CYP3A4 inhibitors—ketoconazole, itraconazole, and posaconazole—were determined and compared to compound-specific hepatic enrichment factors obtained from physiologically based modeling of clinical DDI studies with these three compounds. Kp_uu_ obtained in suspended human hepatocytes gave good predictions of system-dependent differences *in vitro*. The Kp_uu_ was also in fair agreement with the compound-specific hepatic enrichment factors in DDI models and can therefore be used to improve estimations of enrichment factors in physiologically based pharmacokinetic modeling.

**Electronic supplementary material:**

The online version of this article (10.1208/s12248-019-0344-8) contains supplementary material, which is available to authorized users.

## Introduction

Accurate predictions of drug-drug interactions (DDIs) are a challenging task during drug development because the relevant inhibitor concentration is not directly accessible (1,2). Predictions of DDIs depend on *in vitro* parameters such as the half-maximal inhibitory concentration (IC_50_) or the inhibition constant (K_i_) (3). These *in vitro* values are then used in physiologically based pharmacokinetic (PBPK) models to simulate DDIs *in vivo* (4).

For predictions of metabolic DDIs with CYP enzymes, K_i_ or IC_50_ values for a given compound are typically determined in liver microsomes or in hepatocytes. There is a growing interest in using cryopreserved human hepatocytes (HH) instead of human liver microsomes (HLM) for preclinical DDI assessment. In the microsomal setting, the drug concentration available to interact with the enzyme is equal to the unbound drug concentration in the incubation medium (*i.e.*, the nominal incubation concentration, C_inc_, corrected for fraction of unbound drug in the liver microsomes (f_u,mic_)) (5). However, the HH model may be a more physiologically relevant system than HLM, because it takes into account multiple processes that influence enzyme inhibition. In the hepatocyte setting, compounds need to reach the cell interior where the CYP enzymes are located, a process that may involve passive permeability and active transport. Subsequent to this, the intracellular unbound drug concentration (C_u,cell_) may be further influenced by metabolic clearance, protein binding, and partitioning into organelles (*e.g.*, lysosomal trapping) or cellular membranes (5–8). These factors can all contribute to inconsistencies between the C_u,cell_ and C_inc_, leading to differences in the K_i,app_ or IC_50,app_ values measured in HLM and HH. Therefore, careful consideration must be given to which *in vitro* system is appropriate for testing enzyme inhibition.

Determination of C_u,cell_ is especially challenging if active transport is involved and is therefore classified as a low-confidence parameter in PBPK modeling (9). The US Food and Drug Administration (FDA) and the European Medicines Agency (EMA) recommended the [I]/K_i_ ratio (where [I] is the inhibitor concentration) as a parameter in static DDI predictions (10). However, the determination of [I] is not currently standardized. The surrogates commonly used are the average or maximum unbound concentration in blood or at the inlet to the liver, or the maximum total concentration in the circulation at steady state (10–12). In addition to the different methods to estimate [I], the determination of K_i_ also varies. For example, K_i,app_—based on determinations in cryopreserved HH suspended in human plasma—has been proposed (13). Such systems aim to reduce *in vitro* to *in vivo* translational uncertainties by more closely mimicking the *in vivo* situation, building protein binding and intracellular bioavailability considerations into the *in vitro* system.

Strategies for more direct estimations of C_u,cell_ recently emerged (14–18). These include the parallel determination of hepatocellular drug accumulation at steady state (Kp) and the unbound fraction of drug in the cell (f_u,cell_) which are combined to calculate the unbound partition coefficient Kp_uu_. Kp_uu_ is defined as the ratio between unbound drug concentration in the cell interior and the unbound drug concentration in the cell exterior at steady state and can also be derived from kinetic parameters (17).

We recently introduced the term intracellular bioavailability (F_ic_), defined as the ratio between the intracellular unbound concentration, which is available to elicit effects inside the cell, and the extracellular concentration. In the present work, F_ic_ is equal to Kp_uu_, as no protein was added to the incubation media, and therefore, the term Kp_uu_ has been used for simplicity. F_ic_ or Kp_uu_ account for active and passive mechanisms and predict target engagement and phenotypic responses in cells (19). We have shown that this parameter reflects transporter effects in cell lines transfected with single transporters. Further, we have seen differences in the Kp_uu_ values for freshly isolated, plated HH, and suspended HH. This is explained by the altered clearance in the two systems (7).

Given this strong influence of culture formats on Kp_uu_, we hypothesized that Kp_uu_ could be used to reconcile the differences in measurement obtained from different human-relevant experimental systems. We therefore first investigated a series of reference CYP inhibitors (saquinavir, nelfinavir, enoxacin, and clarithromycin) in HLM and HH, previously shown to have differences in K_i,app_ in rat liver microsomes and rat hepatocytes (20,21). Second, we applied an optimized assay for a series of investigational compounds from a drug discovery program that were all identified as CYP2C9 inhibitors. Finally, Kp_uu_ was determined for a series of commonly used CYP inhibitors of the azole antifungal family—ketoconazole, itraconazole, and posaconazole. These compounds were chosen because they require hepatic enrichment factors (referred to as “hepatic uptake value” in the SimCYP software), to reconcile *in vitro* and *in vivo* K_i_ values (11,22). We reasoned that these scaling factors represent Kp_uu_ at the site of DDI.

## Methods

### Chemicals

Compounds were retrieved from the in-house stock at the Roche laboratories at their highest available purity and dissolved at 10 mM in DMSO or, if lower, at their highest solubility. DMSO stocks were kept at − 20°C.

### Compound Selection

The literature was screened for a validation set of compounds with reported discrepancies between cellular and microsomal IC_50_ or Ki values. Enoxacin, clarithromycin, saquinavir, and nelfinavir were identified as suitable candidates (20). The method was then applied to an internal compound set from Roche (hereafter RO compounds) consisting of a structurally related series known to inhibit CYP2C9, and for which information on IC_50_ in HLM and HH was available. As a third compound set, three members of the azole antifungal family—ketoconazole, itraconazole, and posaconazole—were identified as compounds with reported *in vitro* K_i_ values that differ from the K_i_ values reported in models predicting *in vivo* data.

### Cell Culture

A pooled HH batch (BioreclamationIVT, LiverPool 10-donor HH, product no. X008001, Lot RBR) was used in the CYP inhibition assay and for determination drug uptake in suspended HH. After thawing, the cells were suspended in InVitroGro CP medium at the specified concentration in each assay (product no. Z99029, BioreclamationIVT) and used immediately for experiments.

### CYP Inhibition Assay in HLM and HH

For determination of CYP2C9 inhibition, 180 μL of HLM 0.2 mg/mL (BD Gentest, Cat no. 452117, pool from 150 donors of mixed gender) plus substrate (5 μM diclofenac for CYP2C9 and 5 μM midazolam for CYP3A4) was added to a deep 96-well plate together with the test compound or reference inhibitor (concentration range from 0.008 μM to 1 μM). After the addition of 20 μL of 10 mM NADPH, the plate was incubated for 5 min. The reaction was stopped by adding 200 μL of acetonitrile containing an internal standard. After centrifugation of 10 min at 6200×*g*, the concentration of the substrate in the samples was determined by LC-MS (Sciex API4000, see S6 for LC-MS parameters).

For HH, 50 μL of a suspension of cryopreserved HH (3 × 10^6^ cells/mL) were added to a 96-well round bottom plate and pre-incubated for 20 min in Williams E medium (without FBS). Fifty microliters of a × 3 concentrated test compound-solution and 50 μL of × 3 concentrated substrate (diclofenac for CYP2C9, midazolam for CYP3A4 and tacrine for CYP1A2) were added and the plate was incubated on a shaker at 37°C, 5% CO_2_, and 900 rpm. Incubations were stopped after 5 min by transferring 100 μL of the suspension to a fresh plate and adding 100 μL of acetonitrile containing the internal standard and then analyzed as above.

Where available, IC_50_ values were compared to values found in the literature (Table S1).

### Intracellular Unbound Drug Accumulation Ratio (Kp_uu_)

Kp_uu_ was determined as previously described (7,18) using Eq. (1):

1 $$ {\mathrm{Kp}}_{\mathrm{u}\mathrm{u}}=\mathrm{Kp}\bullet \frac{{\mathrm{f}}_{\mathrm{u},\mathrm{cell}}}{{\mathrm{f}}_{\mathrm{u},\mathrm{medium}}} $$

where Kp is the steady-state cellular uptake, and f_u,cell_ the fraction of unbound compound in the cell, determined as described below. The fraction unbound of the compound in the medium (f_u,medium_) was equal to 1 as no serum proteins or other drug binding components were added to the incubation media.

### Steady-State Cellular Uptake (Kp)

The Kp in suspended HH was measured similarly to previous reports with some modifications (7,18). Briefly, cells were rinsed twice with pre-warmed containing 10 mM HEPES. One hundred microliters of cell suspension (5 × 10^6^ cells/mL) was added to a conical 96-well plate, and 100 μL of 1 μM drug solutions in Hank’s buffered salt solution (HBSS) buffered with 10 mM HEPES was added (resulting in a 0.5 μM final concentration). All incubations were performed in triplicates on up to three independent occasions. Full-time curves were established at 1, 3, 7.5, 15, 30, and 45 min to determine Kp at equilibrium. For single time point measurements, the incubation time was matched to the CYP inhibition assays. A sample of the medium was removed after centrifugation in a pre-cooled centrifuge at 4°C (100×*g*, 5 min), and ten-fold diluted with a mixture of acetonitrile and water (60:40) containing the internal standard for determination of C_medium_. The remaining supernatant was removed by aspiration and cells were washed twice with ice-cold buffer. The intracellular compound was extracted using the acetonitrile to water to internal standard mixture for determination of the amount of drug in the cells (A_cell_). Protein content (P_cell_) was quantified using the BCA assay in representative wells to establish the cellular volume (V_cell_), assuming 6.5 μL/mg protein (23). Finally, Kp was calculated using Eq. (2)

2 $$ \mathrm{Kp}=\frac{{\mathrm{A}}_{\mathrm{cell}}/\left({\mathrm{V}}_{\mathrm{cell}\bullet }{\mathrm{P}}_{\mathrm{cell}}\right)}{{\mathrm{C}}_{\mathrm{medium}}} $$

### Determination of f_u,cell_ and f_u,mic_

f_u,cell_ was measured in cassette mode as previously described, but with minor modifications (24). Briefly, frozen cell pellets were thawed on ice and diluted to 10 million cells/mL in HBSS containing 10 mM HEPES. The suspension was homogenized using a mini bead beater (Precellys, EQ02520-300-RD000.0, bead no. VK01) for 2 cycles of 10 s with an intermittent time of 30 s. Compounds were added to the cell homogenate at a final concentration of 0.5 μM and the spiked homogenate was transferred to a dialysis chamber (Rapid Equilibrium Dialysis Device, Thermo Fisher Scientific). HBSS buffered with 10 mM HEPES was placed in the receptor compartment. Samples of spiked cell homogenate were placed at 4 and 37°C for the duration of the experiment for recovery and stability calculations. Incubation time was 4 h at 37°C and 900 rpm for all compounds except itraconazole which reached equilibrium only after 24 h (Fig. S4). The unbound fraction in the cell homogenate (f_u,hom_) was determined according to Eq. (3):

3 $$ {\mathrm{f}}_{\mathrm{u},\hom }=\frac{{\mathrm{C}}_{\mathrm{buffer}}}{{\mathrm{C}}_{\mathrm{hom}}} $$

and the fraction of unbound compound in the cell (f_u,cell_) was calculated by correcting for homogenate dilution according to Eq. (4):

4 $$ {\mathrm{f}}_{\mathrm{u},\mathrm{cell}}=\frac{1}{D\bullet \left(1/{\mathrm{f}}_{\mathrm{u},\hom }-1\right)+1} $$

where the dilution constant *D* was calculated using Eq. (5), and assuming the V_cell_ to be equal to 6.5 μL/mg protein (23). P_hom_ is the protein concentration of the cell homogenate quantified using the BCA assay.

5 $$ D=1/\left({\mathrm{V}}_{\mathrm{cell}}\bullet {\mathrm{P}}_{\mathrm{hom}}\right) $$

The lower detection limit was reached for determination of f_u,cell_ below 0.01%, and the binding was assumed to be linear at the concentrations used, based on previous results (18).

For determination of the fraction unbound to microsomes (f_u,mic_), HLM at 0.2 mg/mL (equivalent concentration to CYP inhibition assay; BD Gentest, Cat no. 452117, pool from 150 donors of mixed gender) were used instead of cell homogenate and calculated in analogy to f_u,hom_ (Eq. 3).

### Determination of Molecular Properties

Molecular properties of literature compounds were determined using ADMET predictor (Simulations Plus, version 7.2) and cross-checked to values published in the public databases DrugBank (25) and PubChem (http://pubchem.ncbi.nlm.nih.gov). Molecular properties (logD, charge, PSA) of RO compounds were calculated using Roche proprietary in silico tools.

### Statistical Analysis

All statistical analyses were performed in Graph-Pad Prism (version 7.04).

## Results

### Method Optimization

**Fig. 1:**
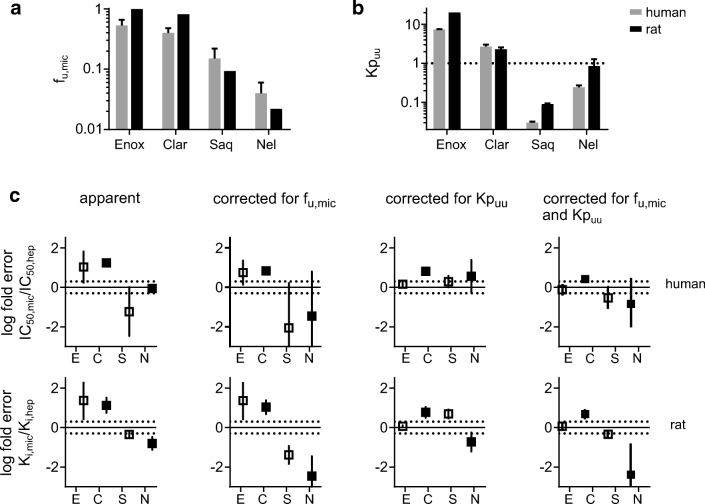
**a** Comparison of f_u,mic_ in human *vs*. rat liver microsomes. Rat values are derived from Brown *et al.* (20). **b** Comparison of Kp_uu_ in human *vs*. rat hepatocytes. **c** Log fold difference of apparent and corrected (unbound) IC_50_ determined in human liver microsomes and human hepatocytes or K_i_ determined in rat microsomes and rat hepatocytes. The dotted lines represent a 2-fold difference. Error bars represent standard deviations. Apparent and corrected K_i_ or IC_50_ values are presented in Tables S1 and S2. Enox, E: enoxacin; Clar, C: clarithromycin; Saq, S: saquinavir; Nel, N: nelfinavir

Four reference compounds of different chemical character were studied first. The physicochemical properties of these drugs are summarized in Table I. Enoxacin and clarithromycin are poorly metabolized, hydrophilic drugs while saquinavir and nelfinavir are extensively metabolized, lipophilic drugs (26). All four have been previously shown to have a clear difference in their K_i,app_ values for liver microsomes and suspended hepatocytes in the rat (Table S1). This made these compounds suitable for investigation in human liver microsomes (HLM) and hepatocytes (HH). The fraction unbound of the four compounds to HLM (f_u,mic_) was determined using equilibrium dialysis and compared to f_u,mic_ in rat liver microsomes (Fig. 1a, Table S3). Kp_uu_ in suspended HH was determined by combining Kp and f_u,cell_ (Table I). Kp_uu_ in rat hepatocytes was derived from Kp values from the literature (Table S3). Enoxacin and clarithromycin had Kp_uu_ values above 1, indicating that they accumulated in HH. In contrast, saquinavir and nelfinavir had Kp_uu_ values below 1 (Fig. 1b).

**Table I:** Major Physicochemical Properties and Results in HLM and Suspended HH. Kp, f_u,cell_, and f_u,mic_ in Rat Liver Microsomes and Hepatocytes Derived from the Literature Are Summarized in S3. Transporter and Enzyme Substrates Are Indicated in Table S7

Compound	MW (g/mol)	Charge	LogD_7.4_	f_u,mic_	f_u,cell_	Kp	Kp_uu_
Enoxacin	320.32	Zwitter	− 1.1	0.53 ± 0.13	1.0 ± 0.0	7.4 ± 1.1	7.4 ± 0.2
Clarithromycin	747.95	Basic	1.6	0.40 ± 0.08	0.10 ± 0.04	25.9 ± 2.1	2.7 ± 0.4
Saquinavir	670.84	Neutral	3.5	0.15 ± 0.07	0.00030 ± 0.00006	109 ± 12	0.030 ± 0.002
Nelfinavir	567.78	Neutral	4.6	0.04 ± 0.02	0.00025 ± 0.00005	956 ± 312	0.24 ± 0.03

Next, we used the f_u,mic_ and Kp_uu_ values in human and rat to scale apparent K_i_ (or IC_50_) values determined in microsomes and hepatocytes, respectively. Before scaling, these apparent values revealed a significant discrepancy between hepatocytes and microsomes, with the differences ranging up to 22-fold (Fig. 1c). To obtain corrected K_i_ or IC_50_, we multiplied apparent K_i_ or IC_50_ in microsomes with f_u,mic_ and apparent K_i_ or IC_50_ in hepatocytes with Kp_uu_ (= Kp × f_u,cell_). After applying these corrections, K_i,app_ or IC_50,app_ values could largely be reconciled (*i.e.*, ~ 2-fold differences). Nelfinavir—the most lipophilic compound in the series—appeared to be an outlier since after correction for f_u,mic_ and Kp_uu_, the difference in K_i_ or IC_50_ increased rather than decreased.

### Kp_uu_ as Scaling Factor of IC_50_ for an RO Discovery Series Inhibiting CYP2C9

**Fig. 2:**
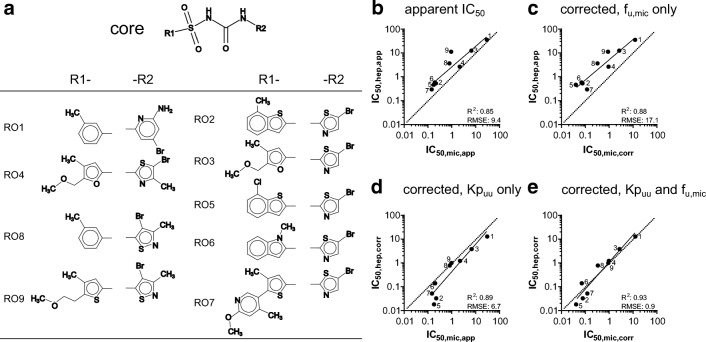
Kp_uu_ as correction factor of IC_50_ in the RO discovery series. **a** Structures of RO discovery compound. **b**–**e** Comparison of IC_50_ values measured in HLM and HH. Hepatocellular IC_50,app_ were corrected with Kp_uu_ (= Kp · f_u,cell_) in suspended HH and microsomal IC_50,app_ values were corrected with f_u,mic_ to obtain IC_50,corr_. The dotted line indicates the line of unity. The labels of the data points correspond to the structure numbers in panel (**a)**

We further applied our approach to an internal compound set (RO compounds). The nine compounds in this discovery series were outliers in the internal screening processes in which IC_50_ values of HH and HLM were compared. The compounds of this series are known to inhibit CYP2C9. These nine compounds are structurally related, with a common core structure containing a sulfonamide and a secondary amide, and aromatic substituents including (iso-)thiazoles, benzothiophenes, pyridines, or furanes (Fig. 2a). They are all acidic at pH 7.4, with logD values indicating hydrophilic properties (Table II).

**Table II:** Properties of the RO Discovery Compound Series and Results in HLM and Suspended HH

Compound	MW (g/mol)	Charge	LogD_7.4_	PSA	pKa	P_app_ (× 10^−6^ cm/s)	f_u,mic_	f_u,cell_	Kp	Kp_uu_
RO01	385.24	Acidic	0.7	123	5.41	10	0.42 ± 0.06	0.0149 ± 0.0003	24.0 ± 2.2	0.357 ± 0.003
RO02	432.34	Acidic	1	153	3.14	2	0.36 ± 0.09	0.00130 ± 0.00007	47.3 ± 1.1	0.0613 ± 0.0002
RO03	410.26	Acidic	− 1	147	2.91	9	0.41 ± 0.03	0.017 ± 0.001	17.9 ± 8.9	0.30 ± 0.08
RO04	424.29	Acidic	− 0.7	147	2.80	13	0.45 ± 0.11	0.02 ± 0.01	27.6 ± 26.1	0.46 ± 0.65
RO05	452.75	Acidic	1.1	153	2.76	2	0.23 ± 0.02	0.0021 ± 0.0003	18.5 ± 4.4	0.039 ± 0.003
RO06	415.29	Acidic	0.4	130	2.80	3	0.36 ± 0.11	0.0039 ± 0.0003	61.6 ± 38.6	0.24 ± 0.10
RO07	503.41	Acidic	0.9	175	3.17	4	0.84 ± 0.06	0.00392 ± 0.00003	41.9 ± 4.8	0.16 ± 0.01
RO08	390.28	Acidic	− 0.1	125	2.66	22	0.41 ± 0.09	0.009 ± 0.004	22.2 ± 4.3	0.21 ± 0.05
RO09	454.38	Acidic	− 0.3	162	2.49	2	0.97 ± 0.41	0.0075 ± 0.0006	11.7 ± 2.0	0.088 ± 0.003

First, we determined the IC_50,app_ in HH and HLM using diclofenac as substrate. The IC_50_ values of RO1, RO3, and RO4 were identical for HH and HLM, while the other compounds differed up to 12-fold (RO9) (Fig. 2b), with, a root-mean-squared error (RMSE) of 9.4 for IC_50,hep,app_ compared to IC_50,mic,app_. As in the previous section, we next determined Kp_uu_ and f_u,mic_ taking care to use the same batches of hepatocytes and microsomes as for the inhibition experiments, respectively (Table II). All parameters were matched to the IC_50_ assay.

We then used the Kp_uu_ and f_u,mic_ values as scaling factors to determine IC_50,corr_. Correction using Kp_uu_ or f_u,mic_ alone did not improve the correlations (Fig. 2c and d). After combining Kp_uu_ and f_u,mic_ to give IC_50,hep,corr_ and IC_50mic,corr_, respectively, the linear correlation between the IC_50_ values improved, giving and *R*^2^ of 0.92 and a 10-fold reduction in RMSE from 9.4 to 0.9 (Fig. 2e).

Because the compounds were analogous to each other, we also analyzed the possible influence of substituents on Kp_uu_. For instance, in five of the nine compounds, the R2 substituent was kept constant while the R1 substituent varied. However, no systematic influence of the change in substituent could be observed. We conclude that the compound series was too small to allow a proper substituent analysis; this underscores the importance of experimental analysis of small series of compounds.

### Kp_uu_ in IVIVE and DDI Models of the Azole Antifungal Family

**Fig. 3:**
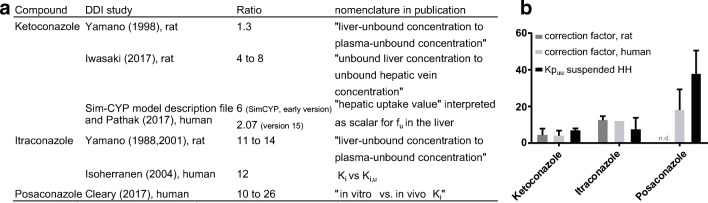
Correlation of IVIVE correction factors with Kp_uu_. **a** Overview of correction factors used for the azole antifungal compounds in *in vivo* DDI studies. **b** Illustration of hepatic uptake scalars in rat and human DDI models (error bars represent range) *vs*. *in vitro* Kp_uu_ in suspended HH (error bars represent SD)

Finally, we investigated if Kp_uu_ could improve predictions by DDI models that are based on *in vitro* K_i_ values. Compounds of the azole family are commonly used as inhibitors of CYP3A4 in preclinical and clinical DDI studies (2,27). A concentrative cellular uptake of ketoconazole, itraconazole, and posaconazole has been widely described, but uptake mechanisms are not fully elucidated (28–31). This concentrative uptake requires correction of K_i_ (which is typically determined in HLM) in order to match *in vivo* K_i_ values in DDI models. Therefore, compound-specific correction factors (referred to as “hepatic enrichment factor” or “hepatic uptake scalar”) have been introduced to improve the IVIVE in several models, which are summarized in Fig. 3a. We therefore investigated if Kp_uu_ values measured in suspended HH agreed with these correction factors (Table III and Fig. 3b). The Kp_uu_ values were high, ranging from 7 to 38, in line with the previously described concentrative uptake. Interestingly, the Kp_uu_ values were within 2-fold of the empirically determined correction factors in the DDI models, indicating that Kp_uu_ is a major contributor to the “hepatic enrichment factor” for the azole series.

**Table III:** Properties and Results for the Azole Antifungal Series and Results in HLM and Suspended HH

Compound	MW (g/mol)	Charge	LogD_7.4_	f_u,mic_	f_u,cell_	Kp	Kp_uu_
Ketoconazole	531.43	Neutral	3.7	0.32 ± 0.12	0.004 ± 0.001	1659 ± 479	6.9 ± 1.1
Itraconazole	705.63	Neutral	4.9	0.21 ± 0.13	0.003 ± 0.002	2717 ± 1573	7.5 ± 6.3
Posaconazole	700.78	Neutral	4.4	0.21 ± 0.11	0.005 ± 0.003	6931 ± 1235	37.6 ± 12.8

## Discussion

In this work, we evaluated the potential of Kp_uu_ as scaling factor for CYP enzyme inhibition studies, using three experimental setups.

For the first set of compounds, we investigated if the determination of intracellular unbound concentrations could be used to reconcile differences in potency in CYP inhibition observed between microsomes and hepatocytes. CYP enzymes can be considered as targets or off-targets facing the cell interior. We have previously shown that F_ic_ or Kp_uu_ can explain differences in potency between isolated intracellular targets in biochemical high-throughput screening assays and the corresponding cellular assays (19). Drug response for intracellular targets in a variety of subcellular compartments including the cytosol, endosomes, and the nucleus has been successfully predicted, using our methodology for determination of F_ic_, F_cyto_, or F_endo_ (19,32). Intracellular unbound drug concentrations are determined by combining the cellular accumulation ratio (Kp) with the fraction of unbound compound in the cell (f_u,cell_). f_u,cell_ is determined using the homogenization method which relies on the assumption that the degree of unspecific binding of drugs is not altered upon homogenization of cells (7,18).

In the first series of experiments, we used reference compounds with previously reported differences in K_i_ of CYPs in rat liver microsomes and rat hepatocytes, as well as Kp, f_u,cell_, and f_u,mic_ values in the rat (20).

Our experimental human f_u,cell_ values differed more than 100-fold for the two lipophilic drugs compared to those in the rat study that was calculated based on lipophilicity and cellular volume (33) (saquinavir 0.14 *vs*. 0.0003 and nelfinavir 0.035 *vs*. 0.0002, rat *vs*. human, respectively, Table S3). There was a better agreement for the two more hydrophilic compounds (enoxacin 0.99 *vs*. 1.00 and clarithromycin 0.86 *vs*. 0.10 in rat *vs*. human, respectively). The homogenization/membrane dialysis method has been compared with the temperature and lipophilicity methods for estimating the f_u,cell_ of a limited series of structural unrelated compounds by Riede *et al.* (15). In their study, the homogenization method gave generally lower values than the temperature and lipophilicity methods. This suggests an overestimation of intracellular binding by the latter two. However, a large variability (up to 13-fold differences) between laboratories has been observed (7,15,17,34,35). f_u,cell_ is dependent on accurate dilution factors for scaling f_u,hom_ to f_u,cell_ (Eq. 4 and 5). In the comparative study by Riede *et al*. (15), very low cell concentrations (130,000 cells/mL) were used, in comparison to 10 million cells/mL in our studies or 50 million cells/mL by Riccardi *et al.* (34,35). Such experimental differences may not allow a reliable comparison between laboratories, but in general, all experimental setups agree on significantly lower f_u,cell_ for highly lipophilic drugs as compared to calculations based on logD. This indicates the importance of using a cellular matrix with relevant lipid and protein binding sites of drugs for the determination of f_u,cell_ (8). Moreover, no significant inter-species variability in f_u,cell_ has been observed in a recent comparative study (34). Therefore, for the four reference compounds, the methodological differences were more significant than the expected inter-species variability in f_u,cell_, and we used our experimental f_u,cell_ values for determination of Kp_uu_ in both rat and human. On the other hand, inter-species variability is expected in Kp values, which can be influenced by active uptake and elimination processes. Therefore, the specific Kp in rat or human hepatocytes was used for determination of Kp_uu_ (Table S3). For the four reference compounds, Kp for the human and rat hepatocytes followed the same rank order and were in the same order of magnitude, with slightly higher values in rat hepatocytes for three out of the four compounds. It should be noted that clarithromycin is a weak base that can be subject to lysosomal trapping. Using pH partitioning theory (18), the calculated cytosolic Kp_uu_ (or F_cyto_) in human hepatocytes would result in 1.3, instead of the experimental value of 2.7, which does not account for subcellular localization of the drug.

Two of the four reference compounds, enoxacin and clarithromycin, displayed a K_i_ ratio (K_i,mic_/K_i,hep_) or IC_50_ ratio and Kp_uu_ above one, indicating a concentrative uptake of the compounds in hepatocytes. By contrast, the K_i_ ratio or IC_50_ ratio and Kp_uu_ were less than one for saquinavir and nelfinavir, suggesting that the lower potency in rat hepatocytes was driven by poor access to the intracellular compartment (and to the CYP enzyme) as opposed to the microsomes where the CYP enzyme is freely exposed in the medium. For three out of the four compounds, K_i_ or IC_50_ values in liver microsomes and hepatocytes were in good agreement after applying relevant Kp_uu_ and f_u,mic_ as scaling factors (Fig. 1c) (34–36). For nelfinavir, scaling K_i_ or IC_50_ values with Kp_uu_ and f_u,mic_ resulted in a larger discrepancy rather than an improvement. Interestingly, nelfinavir was identified as an outlier also in our previous Kp_uu_ studies in MDCK cells, suggesting the involvement of unknown active processes that are not captured by the Kp_uu_ methodology (7). Nelfinavir is very lipophilic, poorly soluble, and a substrate of active efflux (P-gp)—these properties suggest that the compound is prone to nonspecific binding and that there are significant confounding effects occurring in the hepatocyte experiment. Indeed, poor mass balance (~ 50%) was observed for nelfinavir and the similarly lipophilic saquinavir in the hepatocyte experiments. As the mass balance was recovered in the presence of the CYP inhibitor ABT, this confirms that the loss of compound is due to hepatic metabolism (Fig. S4). The hepatocyte metabolism of nelfinavir and saquinavir has been reported previously in a study that identified these two compounds as outliers. This was based on their intrinsic clearance in microsomes being much higher than in hepatocytes, compared to other compounds with similar clearance mechanisms (37).

It should be noted, however, that in our studies, the hepatocyte metabolism of nelfinavir and saquinavir was not a limiting factor for establishing the concentration equilibrium, since Kp values were unaffected by the presence or absence of ABT (Fig. S4). Furthermore, the use of albumin or an increase in temperature of wash buffers from 4 to 37°C (38,39) did not change the results significantly (Fig. S5), which precludes the likelihood that non-specific binding was affecting the results. Thus, the discrepancy in the K_i,corr_ values of nelfinavir could not be explained by CYP-mediated metabolism or non-specific binding and is likely driven by other factors. Despite the discrepancy for nelfinavir, we overall obtained a significant harmonization of the K_i_ ratios between microsomes and hepatocytes, suggesting the validity of this approach and indicating the value of further elaboration.

In the next set of experiments, we therefore harmonized the experimental conditions between the CYP inhibition experiments and the Kp_uu_ experiments. Instead of comparing Kp_uu_ to CYP inhibition constants from the literature, we determined IC_50_ in HH and HLM with methodologies as similar as possible to each other to generate a consistent dataset. As a result, the Kp_uu_- and f_u,mic_-corrected IC_50_ values for the discovery series of CYP2C9 enzyme inhibitors were in excellent agreement for HH and HLM (Fig. 2e). We conclude that the best agreement between the two systems is obtained by applying simultaneous correction factors for both systems, along with taking into consideration differences in assay setup and inter-batch variability.

Given the significance of Kp_uu_ in the two series of *in vitro* experiments, we next evaluated if it could also be used as a scaling factor in *in vivo* studies. More specifically, we investigated if Kp_uu_ could be used to estimate the empirical hepatic uptake scalar required in PBPK models of ketoconazole, itraconazole, and posaconazole as CYP inhibitors. Many models use such empirically determined scalars (Fig. 3a). However, others avoid them because the uptake mechanism of these azole compounds are not mediated via classic hepatic uptake transporters such as the OATP or OCT transporter family (29,40). Kp_uu_ is a measure of all combined effects in a cell and provides a mechanistic tool for estimation of uptake scalars without the need for elucidating the underlying factors contributing to the net drug disposition in the cell. We found that Kp_uu_ values in suspended HH were in good agreement with the empirically determined hepatic uptake scalars in the models for both rat and human (Fig. 3). In the case of itraconazole, the three major metabolites are equally or more potent inhibitors of CYP3A4 than the parent compound, which is a complicating factor in PBPK modeling (28,40,41). The measurement of Kp_uu_ of the parent compound alone was however sufficient for recovering the hepatic uptake scalars. Therefore, we believe that Kp_uu_ can be rationally integrated into prospective PBPK modeling approaches. The use of HH instead of its rat counterpart reduces the potential effects of inter-species differences in drug transporter expression and metabolic enzyme sensitivity to inhibition. It is also the most relevant *in vivo* model system for making extrapolations to humans. The relevance of Kp_uu_ to *in vivo* studies is further supported by a recent study in which a series of four statins was assessed for *in vitro* and *in vivo* liver enrichment (35); *in vivo* liver-to-plasma, Kp_uu_ in rat and human were in good agreement with *in vitro* Kp_uu_ obtained in cryopreserved suspended hepatocytes. Similar scenarios are anticipated for, *e.g.*, rifampicin, which has an empirical hepatic uptake scalar of 16.9 (42) and for bosentan, with a hepatic uptake scalar of 5 to 6 (43).

**Fig. 4:**
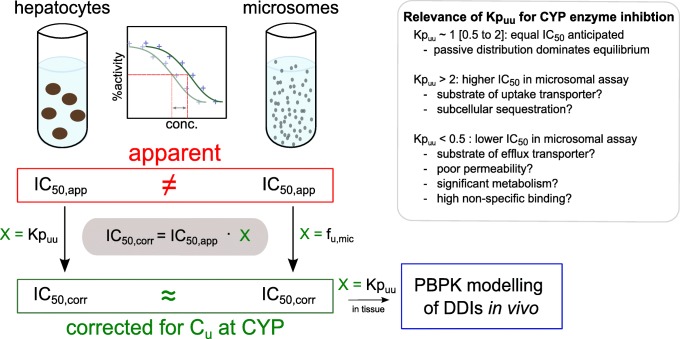
Summary of use and interpretation of Kp_uu_ in the context of CYP-mediated DDI

Determination of K_i,app_ directly in HH can be an alternative to the incorporation of hepatic uptake scalars. However, most K_i_ screens are based on HLM. To make our approach more generally applicable, we suggest the use of K_i,corr_ values, and account for intracellular unbound concentrations in the target cells in any tissue of interest (Fig. 4). Our approach is not limited to the liver and can be applied on any cell of interest with an intracellular target, including studies of DDIs in intestinal enterocytes.

## Conclusion

In summary, our results indicate that Kp_uu_ in HH is an easy-to-interpret *in vitro* parameter that can be used as a human-relevant scaling factor to bridge the differences of experimental systems such as liver microsomes and hepatocytes and gives a mechanistic understanding of any result discrepancies (Fig. 4). Since Kp_uu_ is the net result of all processes that affect the intracellular unbound drug concentration in HH, no prior knowledge of uptake and elimination processes is required. Indeed, very high or low Kp_uu_ values can indicate that active cellular processes are playing a significant role in drug disposition. Therefore, Kp_uu_ can be used as a decision tool (*e.g.*, if the Kp_uu_ is lower than 0.5 or higher than 2) for more detailed investigations. Very lipophilic compounds have been found to be more challenging for assessment of Kp_uu_ and future work should focus on this compound class. Further, Kp_uu_ has been shown to reflect hepatic uptake scalars used in literature for the triazole antifungal family and we believe that the use of Kp_uu_ is an approach that can be pursued to provide a mechanistic understanding of scalars used in PBPK models that predict drug exposure and DDI potential.

## Electronic Supplementary Material

ESM 1 (PDF 970 kb)
