# Supplementary material for: Impact of Intracellular Concentrations on Metabolic Drug-Drug Interaction Studies
Source: AAPS J. 2019 Jun 18;21(5):77. doi: 10.1208/s12248-019-0344-8 (PMC6581936; doi:10.1208/s12248-019-0344-8)
Supplement: Supplementary file 1 — (PDF 970 kb) [file 12248_2019_344_MOESM1_ESM.pdf]

Supplementary material

Impact of Intracellular Concentrations on Metabolic Drug-Drug Interaction Studies

Treyer, Andrea[1]; Ullah, Mohammed[2]; Parrott, Neil[2]; Molitor, Birgit[2]; Fowler, Stephen[2]; Artursson, Per[1,3,4]*

Content:

S 1 Collection of IC_{50} and K_i values

S 2 Apparent and corrected IC_{50} or K_i values

S 3 Rat vs. human Kp, $f_{u,cell}$ and $f_{u,mic}$

S 4 Kp in presence of metabolic inhibitor ABT

S 5 Use of albumin and influence of temperature of washing buffers

S 6 Dialysis equilibrium time

S 7 Transporter and enzyme interactions of compounds

S 8 LC-MS parameters

Supplementary References

1. Emoto, C., et al., *In vitro inhibitory effect of 1-aminobenzotriazole on drug oxidations in human liver microsomes: a comparison with SKF-525A.* Drug Metab Pharmacokinet, 2005. **20**(5): p. 351-7.
2. Parker, A.J. and J.B. Houston, *Rate-limiting steps in hepatic drug clearance: comparison of hepatocellular uptake and metabolism with microsomal metabolism of saquinavir, nelfinavir, and ritonavir.* Drug Metab Dispos, 2008. **36**(7): p. 1375-84.
3. Brown, H.S., et al., *Comparative use of isolated hepatocytes and hepatic microsomes for cytochrome P450 inhibition studies: transporter-enzyme interplay.* Drug Metab Dispos, 2010. **38**(12): p. 2139-46.
4. Treyer, A., et al., *Intracellular Drug Bioavailability: Effect of Neutral Lipids and Phospholipids.* Mol Pharm, 2018. **15**(6): p. 2224-2233.
5. Mateus, A., P. Matsson, and P. Artursson, *Rapid measurement of intracellular unbound drug concentrations.* Mol Pharm, 2013. **10**(6): p. 2467-78.
6. Mateus, A., et al., *Intracellular drug bioavailability: a new predictor of system dependent drug disposition.* Sci Rep, 2017. **7**: p. 43047.
7. Templeton, I.E., et al., *Contribution of itraconazole metabolites to inhibition of CYP3A4 in vivo.* Clin Pharmacol Ther, 2008. **83**(1): p. 77-85.
8. Riccardi, K., et al., *Plasma Protein Binding of Challenging Compounds.* J Pharm Sci, 2015. **104**(8): p. 2627-36.
9. Law, V., et al., *DrugBank 4.0: shedding new light on drug metabolism.* Nucleic Acids Res, 2014. **42**(Database issue): p. D1091-7.

S 1 Collection of IC_{50} and K_i values

Inhibitor	CYP	Substrate/Metabolite	System	IC_{50} or K_i* [µM]	Source
Enoxacin	1A2	TPH/1,3-DMU	HLM	2140 ± 370	[1]
		TPH/1,3-DMU	RLM	2800 ± 1200*	[2]
		TCR/1’-OH	HH	422 ± 93	[3]
		TPH/1,3-DMU	RH	120 ± 65*	[2]
Clarithromycin	3A4	MDZ/1’-OH	HLM	300	[4]
		MDZ/1’-OH	RLM	1000 ± 100*	[2]
		MDZ/1’-OH	HH	19.21	[3]
		MDZ/1’-OH	RH	75 ± 24 *	[2]
Saquinavir	3A4	MDZ/1’-OH	HLM	1.2 ± 0.4	[5]
		MDZ/1’-OH	HLM	0.7	[6]
		MDZ/1’-OH	HLM	1.1	[3]
		MDZ/1’-OH	RLM	0.22 ±0.07*	[2]
		MDZ/1’-OH	HH	29 ± 16	[5]
		MDZ/1’-OH	HH	5.4	[3]
		MDZ/1’-OH	RH	0.5 ± 0.035*	[2]
Nelfinavir	4A4	MDZ/1’-OH	HLM	1.3 ± 0.6	[5]
		MDZ/1’-OH	HLM	8.0	[6]
		MDZ/1’-OH	HLM	0.63	[3]
		MDZ/1’-OH	RLM	0.46 ±0.1*	[2]
		MDZ/1’-OH	HH	5.9 ± 0.7	[5]
		MDZ/1’-OH	HH	1.7	[3]
		MDZ/1’-OH	RH	2.94 ± 1.02*	[2]

TPH: Theophylline; 1,3-DMU: 1,3-Dimethyluric acid; TCR: Tacrine; MDZ: Midazolam; 1’-OH: 1'-hydroxy metabolite; HLM/RLM: human/rat liver microsomes; HH/RH: human/rat hepatocytes

[1] Niki et al., Antimicrob Agents Chemother. 1998 Jul;42(7):1751-5

[2] Brown et al., Drug Metab Dispos 2010, 38(12): 2139-2146

[3] In house

[4] Gascon & Dayer ; Eur J Clin Pharmacol 1990 41(6): 573-578

[5] Rioux et al., Xenobiotica. 2013 Jul;43(7):592-7

[6] Fahmi et al., Drug Metab Dispos. 2008 Aug;36(8):1698-708

S 2 Apparent and corrected IC_{50} or K_i values

Human hepatocytes and liver microsomes

	Kp_{uu}	$f_{u,mic}$	$IC_{50,app}$ *		$IC_{50,corr}$ **	
			HH	HLM	HH	HLM
Enoxacin	7.37 ± 0.16	0.53 ± 0.13	422 ± 93	2140 ± 370	3110.1 ± 688.7	1134.2 ± 340.4
Clarithromycin	2.67 ± 0.35	0.4 ± 0.08	19.21 ± 0	330 ± 0	51.3 ± 6.7	132 ± 26.4
Saquinavir	0.03 ± 0.002	0.15 ± 0.07	17.2 ± 16.7	1 ± 0.26	0.52 ± 0.5	0.15 ± 0.08
Nelfinavir	0.24 ± 0.03	0.04 ± 0.02	3.8 ± 3	3.31 ± 4.1	0.91 ± 0.73	0.13 ± 0.18

*average values from table S1

**HH: $IC_{50,corr} = IC_{50,app} \times Kp_{uu}$; HLM: $IC_{50,corr} = IC_{50,app} \times f_{u,mic}$

Rat hepatocytes and microsomes

	Kp_{uu}	$f_{u,mic}$*	$K_{i,app}$*		$K_{i,corr}$ **	
			RH	RLM	RH	RLM
Enoxacin	20 ± 0	0.99	120 ± 65	2800 ± 1200	2400 ± 1300	2772 ± 1188
Clarithromycin	2.27 ± 0.28	0.81	75 ± 24	1000 ± 100	170.3 ± 58.4	810 ± 81
Saquinavir	0.09 ± 0.003	0.093	0.50 ± 0.035	0.22 ± 0.07	0.045 ± 0.003	0.02 ± 0.007
Nelfinavir	0.84 ± 0.43	0.022	2.94 ± 1.02	0.46 ± 0.1	2.5 ± 1.5	0.01 ± 0.002

* Brown et al., Drug Metab Dispos 2010, 38(12): 2139-2146

**RH: $IC_{50,corr} = IC_{50,app} \times Kp_{uu}$; RLM: $IC_{50,corr} = IC_{50,app} \times f_{u,mic}$

S 3 Rat vs. human Kp, $f_{u,cell}$ and $f_{u,mic}$

	Rat (Brown et al, DMD, 2010)		
	Kp	$f_{u,cell}$*	$f_{u,mic}$
Enoxacin	20	0.99	0.99
Clarithromycin	22	0.86	0.81
Saquinavir	306	0.14	0.093
Nelfinavir	3352	0.035	0.022

*calculated from Kilford et al. (2008)

	Human		
	Kp	$f_{u,cell}$*	$f_{u,mic}$
Enoxacin	7.4 ± 1.1	1.0 ± 0.0	0.53 ± 0.13
Clarithromycin	25.9 ± 2.1	0.10 ± 0.04	0.40 ± 0.08
Saquinavir	109 ± 12	0.00030 ± 0.00006	0.15 ± 0.07
Nelfinavir	956 ± 312	0.00025 ± 0.00005	0.04 ± 0.02

*homogenization method/equilibrium dialysis

S 4 Kp in presence of metabolic inhibitor ABT

A batch of HH derived from a single donor and a pooled batch were each used to measure Kp at 45 minutes in presence and absence of the CYP inhibitor ABT.[1] Clarithromycin and enoxacin have comparably low metabolic turnover rates and were stable over the investigated timeframe (mass balance ~100%). The mass balance of nelfinavir and saquinavir dropped to ~20-50% due to high metabolic turnover rates [2, 3]. Upon addition of ABT, the mass balance of saquinavir and nelfinavir approximately doubled. Despite this increase in mass balance, similar Kp values were observed in all conditions.

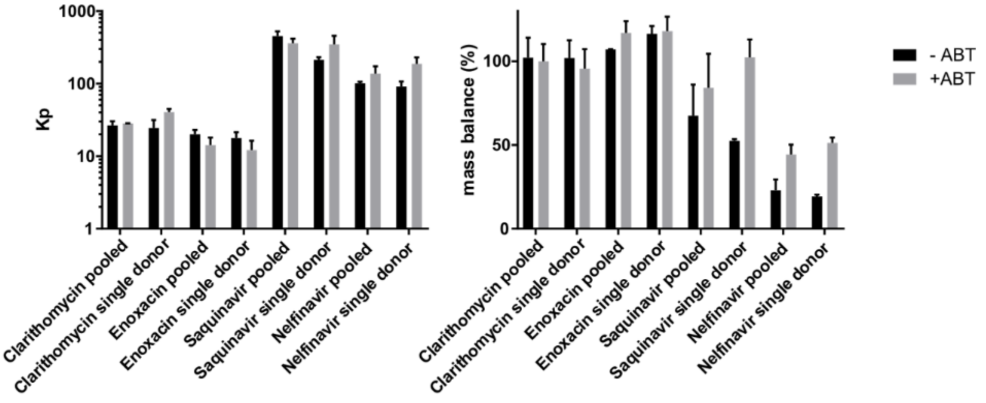

Time curves over 45 minutes were established in plated HH. All compounds reached equilibrium after 5-15 min (Figure A), which is in agreement with previous findings.[4, 5] Also here, the mass balance of nelfinavir and saquinavir increased significantly upon addition of ABT (*p<0.05, n=3, unpaired t-test). Overall, the mass balance was higher in plated hepatocytes than in suspended hepatocytes. This was in-line with our previous findings where metabolic clearance was higher in freshly isolated suspended hepatocytes than in the same cells cultivated in monolayers.[6]

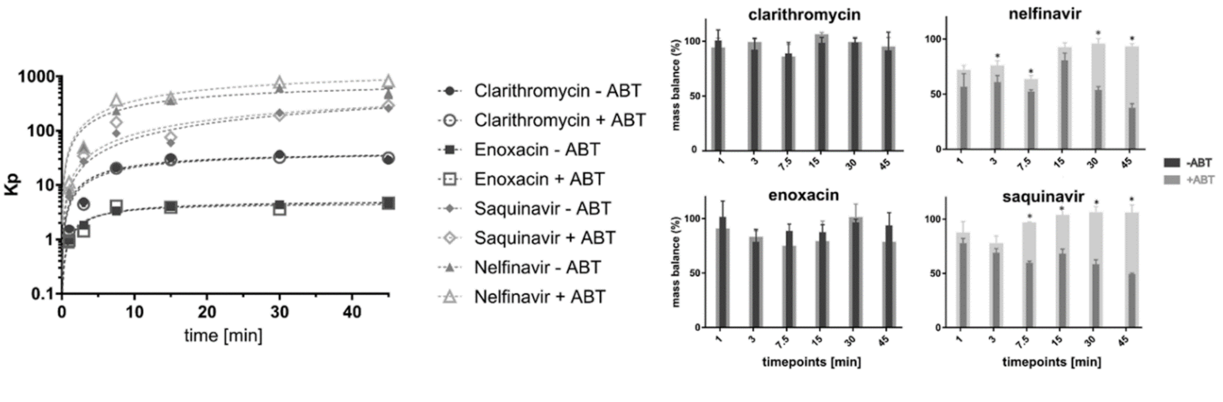

S 5 Use of albumin and influence of temperature of washing buffers

We determined the Kp_{uu} of nelfinavir, the most lipophilic drug in the literature compound series, using buffers at 4 and 37°C, and with and without albumin. The resulting Kp_{uu} values did not differ significantly, except for the comparison of buffer at 4°C without albumin vs. the buffer at 37°C containing albumin — presumably due to higher back-flux of the drug to the medium (one-way ANOVA, Holm-Sidak's multiple comparison test, $p>0.05$).

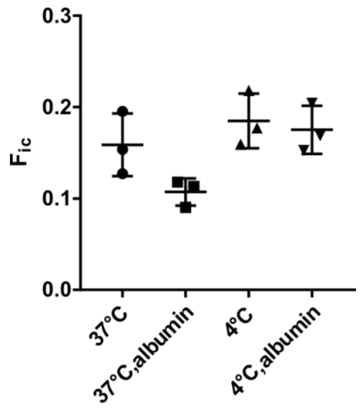

Figure S1: Triplicate measurement at 45 minutes, under four different buffer conditions.

S 6 Dialysis equilibrium time

$f_{u,cell}$ (panel A) and $f_{u,mic}$ (panel B) was measured over 4 to 24 h for ketoconazole, posaconazole, itraconazole and the laboratory standards atorvastatin and lopinavir. Equilibrium was reached within 4h dialysis time for all compounds tested, with the exception of itraconazole. High nonspecific binding has been reported before for this compound [7, 8] explained by its high molecular weight, high lipophilicity and resulting low solubility. The dialysis time for itraconazole used in this study was 24h.

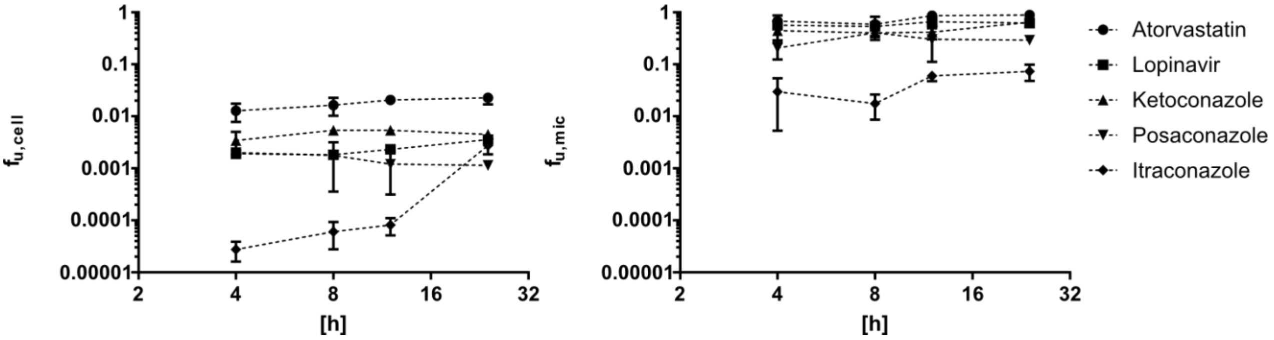

S 7 Transporter and enzyme interactions of compounds

**Compound**	**OATP1A2**	**OATP1B1**	**OATP1B3**	**OATP2B1**	**P-gp**	**MRP1,2**	**BCRP**	**BSEP**	**OAT2**	**OCT1**	**CYP1A2**	**CYP3A4**	**CYP3A5**	**CYP2D6**	**CYP2B6**	**CYP2C9**	**CYP2C19**	**CYP11A1**	**CYP3A7**	**CYP2C8**
Enoxacin	●				●						○									
Clarithromycin					●○			●	○		○	●○	●○				●		●	
Saquinavir		○		○	●○	●		●		○		●○	●○	●○				●	●○	○
Nelfinavir	○	○	○	○	●○		○	●		○	○	●○	○	○	○	○	●○		●○	

Information retrieved from DrugBank data base [9]

● substrate ○ inhibitor

Name	Inhibitor of	Victim drug
Enoxacin	CYP1A2	Theophylline, Tacrine
Clarithromycin	CYP3A4	Midazolam
Nelfinavir	CYP3A4	Midazolam
Saquinavir	CYP3A4	Midazolam
RO1	CYP2C9	Diclofenac
RO2	CYP2C9	Diclofenac
RO3	CYP2C9	Diclofenac
RO4	CYP2C9	Diclofenac
RO5	CYP2C9	Diclofenac
RO6	CYP2C9	Diclofenac
RO7	CYP2C9	Diclofenac
RO8	CYP2C9	Diclofenac
RO9	CYP2C9	Diclofenac
Ketoconazole	CYP3A4	Midazolam, Nifedipine
Posaconazole	CYP3A4	Alectinib
Itraconazole	CYP3A4	Midazolam

S 8 LC-MS parameters

The HPLC system consisted of 20AD Shimadzu pumps. The analytical column was a 2cm × 2.1mm Supelco Ascentis Express C18 with 2.7µm particle size at 60°C. A SCiex Qtrap6500 mass spectrometer equipped with a TurboIonSpray source (IonSpray Voltage in positive or negative mode) and a HTS CTC PAL autosampler were used. Mobile phase A was formic acid 0.5% in water:methanol 95:5, mobile phase B was acetonitrile. 1µl aliquots of the sample solutions were injected and transferred at the analytical column at a flow rate of 600µL/min. To elute the compounds, a high pressure linear gradient from 0% to 95% B in 40 seconds was applied. Total run time was 1.6 min.

Compound	Parent m/z	Daughter Ion m/z	RT [min]	Ionization Voltage	Collision Energy
Enoxacin	321.2	232.1	0.53	5500	50
Clarithromycin	748.5	158.1	0.77	5500	45
Nelfinavir	568.2	135.1	0.77	5500	71
Saquinavir	671.5	570.4	0.78	5500	43
RO1	384.9	214.1	0.76	5500	40
RO2	431.7	147	0.92	5500	39
RO3	407.8	204	0.77	-4500	-20
RO4	421.9	204.2	0.85	-4500	-20
RO5	449.7	245.9	0.85	-4500	-24
RO6	412.8	208.9	0.86	-4500	-22
RO7	502.7	325	0.92	5500	19
RO8	390.1	192.7	0.86	5500	40
RO9	454.1	192.9	0.82	5500	40
Ketoconazole	530.9	489.1	0.76	5500	41
Posaconazole	701.2	683.3	0.88	5500	45
Itraconazole	705.2	392.2	0.94	5500	49
